# Metal and Metal Oxides Nanoparticles and Nanosystems in Anticancer and Antiviral Theragnostic Agents

**DOI:** 10.3390/pharmaceutics15041181

**Published:** 2023-04-07

**Authors:** Tatyana I. Shabatina, Olga I. Vernaya, Nikolay L. Shimanovskiy, Mikhail Ya. Melnikov

**Affiliations:** 1 Department of Chemistry, M.V. Lomonosov Moscow State University, Leninskie Gori Build. 1/3, Moscow 119991, Russia; 2 Faculty of Fundamental Sciences, N.E. Bauman Moscow Technical University, Moscow 105005, Russia; 3 Department of Molecular Pharmacology and Radiobiology, N.I. Pirogov Russian National Research Medical University, Moscow 117997, Russia

**Keywords:** iron oxides, nanoparticles, ferrites, magnetic hyperthermia, magnetic resonance imaging, drug delivery, theragnostic agents of cancer, antiviral therapy, silver, gold, photodynamic therapy, photo-thermal therapy

## Abstract

The development of antiviral treatment and anticancer theragnostic agents in recent decades has been associated with nanotechnologies, and primarily with inorganic nanoparticles (INPs) of metal and metal oxides. The large specific surface area and its high activity make it easy to functionalize INPs with various coatings (to increase their stability and reduce toxicity), specific agents (allowing retention of INPs in the affected organ or tissue), and drug molecules (for antitumor and antiviral therapy). The ability of magnetic nanoparticles (MNPs) of iron oxides and ferrites to enhance proton relaxation in specific tissues and serve as magnetic resonance imaging contrast agents is one of the most promising applications of nanomedicine. Activation of MNPs during hyperthermia by an external alternating magnetic field is a promising method for targeted cancer therapy. As therapeutic tools, INPs are promising carriers for targeted delivery of pharmaceuticals (either anticancer or antiviral) via magnetic drug targeting (in case of MNPs), passive or active (by attaching high affinity ligands) targeting. The plasmonic properties of Au nanoparticles (NPs) and their application for plasmonic photothermal and photodynamic therapies have been extensively explored recently in tumor treatment. The Ag NPs alone and in combination with antiviral medicines reveal new possibilities in antiviral therapy. The prospects and possibilities of INPs in relation to magnetic hyperthermia, plasmonic photothermal and photodynamic therapies, magnetic resonance imaging, targeted delivery in the framework of antitumor theragnostic and antiviral therapy are presented in this review.

## 1. Introduction

Nowadays metal and metal oxides nanoparticles (NPs) and nanosystems (NS) have found numerous applications and play an essential role in modern biomedicine. Nanoparticles are the ultrafine objects that combine atoms of chemical elements or molecules of organic and inorganic compounds with sizes of 1–100 nanometers (nm; 1 nm = 10^−9^ m) [1]. The word nano is derived from the Greek word “nanos”, which means small [2]. Nanoparticles are characterized by a high surface area-to-volume ratio, enormous reactive surface area and their properties are significantly influenced by both classical and quantum effects. Hybrid NSs based on metal and metal oxide NPs also ordinarily include functionalized organic ligands that can interact with surface active centers of the metal/metal oxide NPs and form highly ordered organic coronas around the inorganic cores. These coronas protect them against the effects of biological media, bioactive (drug) substances that can be immobilized directly on the surfaces of metal/metal oxides NPs or incorporated into the soft organic corona structures. Consequently, these materials have unique biological, physical, and chemical properties unlike macro-scale (bulk) materials with the same content [3]. Metallic NPs, such as Ag NPs and Au NPs, have been of significant interest in the past decades due their therapeutic (antibacterial, anticancer, antiviral) and unique physicochemical properties (surface plasmon resonance (SPR), laser and microwave hyperthermia) properties. The combination of therapeutic effects of metal/metal oxides NPs with modern antibacterial, anticancer, antiviral drug molecules can lead to the appearance of a synergetic rise in biomedical efficiency of such hybrid medical formulations. Magnetic nanoparticles (MNPs) of magnetite, maghemite and ferrites could possess ferromagnetic, ferrimagnetic and superparamagnetic properties. They exhibit a high value of the saturation field, increased anisotropy contributions, high field irreversibility, shifted loops after field cooling, and superparamagnetic properties. These features result from the limited and narrow size distribution and surface impacts that determine the magnetic behavior of each nanoparticle [4]. Inorganic nanoparticles (INPs) have shown high potential as targeted nanocarriers for therapeutic, diagnostic, and clinical medications [5]. Their unique physicochemical features are effective at both the molecular and cellular levels.

As per GLOBOCAN 2020, cancer lethality almost reached 10 million in 2020 with 19.3 million new cases diagnosed and up to 28.4 million cases predicted for 2040 [6]. Thus, new theragnostic approaches to the diagnosis and medicine treatment of cancer are required. Among various medical applications, INPs of metal and metal oxides have a special potential in cancer diagnostic and therapy. As a result of intensive research and preclinical investment in liposomal based drug delivery systems, several products with improved cancer treatment potential were produced and approved by the Food and Drug Administration of United States [3,7]. Furthermore, the marketing and clinical application of INPs-based medicines includes Ferumoxsil (Lumirem/Gastromark) and Ferumoxytol (Rienso/Feraheme) [8,9,10]. Regardless, the possibilities of nanomedicine and nanodiagnostics have not been exhausted.

Infectious diseases, along with cancer, are the leading cause of death worldwide. They have a global impact on world health and socio-economic status. COVID-19 is a good example of this [11]. The development of novel treatment strategies for viral diseases is therefore required.

Currently, there are numerous proposed and developed applications of inorganic nanoparticles (INPs) for the diagnosis and treatment of cancer, as well as for antiviral therapy. Most of these applications are still in the early stages of preclinical evaluation. The use of nanotechnology plays a crucial role in tumor diagnostics, with MNPs of metals and their oxides proving to be promising agents for magnetic resonance imaging(MRI). It is predicted that these NPs will replace the more harmful gadolinium compounds in near future. The use of MNPs in cancer treatment has yielded positive results, particularly when it comes to magnetic hyperthermia (MHT), which increases thermal therapy’s effectiveness without the need for surgery. Plasmonic nanomaterials offer another non-invasive form of selective thermal therapy, photothermal therapy (PTT). Photosensitizers for photodynamic (PDT) cancer therapy often include metal and metal oxide NPs. The large specific surface area of INPs, which can be modified and loaded with medicinal substances, provides the basis for the creation of targeted delivery systems for antiviral and antitumor drugs. Surface modification also holds promise in reducing toxicity and increasing the bioavailability of diagnostic and drug systems based on INPs. Ultimately, the purpose of this review is to emphasize the main possibilities of INPs in the field of cancer theragnostic and antiviral treatment.

## 2. Applications of MNPs for MRI in Cancer Diagnostics

Early diagnosis is one of the key parameters that determine the effectiveness and positive outcome of any type of therapy. Detection of stage 1 cancers is associated with a higher than 90% 5-year survival rate [12] due to availability of curative treatment. Medical imaging technologies have undergone explosive growth over the past few decades and now play a central role in clinical oncology.

Magnetic resonance imaging (MRI) has become one of the most widely used and powerful tools for noninvasive clinical cancer diagnostics due to the high degree of soft tissue contrast, spatial resolution and penetration depth compared to other methods. The disadvantage of MRI is its low sensitivity and specificity (false positive rate of 8% for breast cancer) [13]. Contrast agents to some extent allow researchers to improve the MRI sensitivity. The potential and efficiency of MRI contrast agents could be corrected by using cellular markers and ferrimagnetic and superparamagnetic NPs.

### 2.1. MRI Contrast Agents

The use of contrast agents in MRI results in a change of the relaxation times (T_1_ and T_2_) values of water protons and, consequently, a change of signal strength in different body parts where the agent is presented. As a result, the sensitivity and specificity of MRI images of different tissues and organs increases. The MRI contrast agents usually reduce the rate of all relaxation processes; however, each substance predominantly affects one of them. There are two categories of contrast agents in MRI. Positive contrast agents, or T_1_-contrast agents, tend to decrease the relaxation time of the longitudinal component of the magnetization. On the other hand, negative contrast agents, or T_2_-contrast agents, primarily decrease the relaxation time of the transverse component [14]. Enhanced contrast occurs when a tissue exhibits greater affinity for contrast agents or higher vascularity compared to others. Tumors and other diseased tissues differ metabolically from healthy ones, leading to varied absorption of contrast agents and dissimilar MRI images.

### 2.2. T_1_-Contrast Agents

Organic compounds containing metal ions, such as gadolinium (Gd(III)), iron (Fe(III)), and manganese (Mn(II)), make up paramagnetic T_1_ contrast agents. The majority of the available MRI contrast agents are gadolinium chelates which elicit a “positive contrast” or an amplified signal on T_1_-weighted images, while their impact on T_2_-weighted images is usually insignificant [15]. In addition to the effectiveness of free gadolinium ions (Gd^3+^) as a contrast agent due to good T_1_-weighted imaging, these ions also cause significant side effects. The Gd^3+^ ions are rather toxic and induce disturbances in the functioning of all systems: cardiovascular, gastrointestinal, nervous systems, respiratory system, skin, special senses (lacrimation, eye pain and irritation, conjunctivitis, ear pain, taste abnormality, and dry mouth) [15]. Therefore, it is necessary to administer gadolinium in the form that prevents the release of the metal ion in vivo, such as of stable chelate complexes. Unfortunately, this form is not efficient enough [16]. Gadolinium compounds may cause nephrogenic systemic sclerosis [17], neurotoxicity [18]. Histopathological and molecular analysis showed damage in the liver, lungs, and kidney tissues of mice treated by gadolinium-based contrast agents [19]. For that reason, a general warning has been issued by the Food and Drug Administration (United States) for all gadolinium-based contrast agents. Moreover, they advised that gadolinium-based contrast agents should not be used on all patients with acute renal insufficiency [20].

Studies on both animals and humans have revealed that Gd^3+^ (either free or chelated) can persist in the tissues of individuals with normal kidney function. Gadolinium has been detected in several organs, such as the skin, bones, and brain, for years after linear or macrocyclic administering of chelated gadolinium. Nevertheless, the implications of this occurrence remain unclear [21].

Contrast agents based on Gd are typically not specific and tend to rapidly escape from the vascular space due to their molecular weight being low. After intravenous injection, they rapidly move from the blood pool to the interstitial space, exhibiting a distribution half-life (t_1/2_) of approximately 5 min. These agents are primarily excreted by the kidneys, with an elimination t_1/2_ of approximately 80 min [22]. The constrained resolution of the imaging system causes a decrease in visible activity of small areas or objects [23]. Consequently, different varieties of MRI contrast agents were utilized because of the inadequate detection sensitivity, toxicity, and short blood circulation times associated with T_1_ Gd-based contrast agents.

### 2.3. T_2_-Contrast Agents

The MR signal intensity is decreased by the negative (T_2_) contrast agents in the body parts where they are located. The most common and the first NPs used as predominantly negative (T_2_) MRI agents are the magnetic iron oxide nanoparticles (MIONPs). The MIONPs are monocrystalline and consist of maghemite (γ-Fe_2_O_3_) or magnetite (Fe_3_O_4_). Depending on the size of the nanoparticle core, these NPs can be either superparamagnetic or ferromagnetic. Superparamagnetic behavior is typical for particles smaller than 25–35 nm [24]. Iron oxide-based contrast agents are considered to have better biocompatibility and safety profiles when compared to Gd-based agents due to the essentiality of iron in the human body unlike gadolinium. The T_2_ contrast agents result in regions appearing darker in T_2_-weighted images, as they produce hypo intense signals. This phenomenon may be attributed to the heterogeneity of the magnetic field enveloping the NP. Water molecules diffuse through this field, which then leads to dephasing of the magnetic moments of the protons and leads to a shortening of T_2_.

The MIONPs exhibit excellent biomedical capabilities, primarily as MRI contrast agents, due to their high proportion of surface atoms, biodegradability, low toxicity, chemical stability in physiological conditions, and rapid response to external magnetic field stimulation. They have been used as T_2_ contrast agents for more than three decades since 1987. Various compounds that include superparamagnetic iron oxide are commercially available, such as Resovist (Schering, Berlin, Germany; Osaka, Japan), Endorem (Guerbet, Villepinte, France), and Feridex (Berlex, Hanover, NJ, USA). These compounds are typically utilized for detecting liver and spleen tumors [25]. Currently, new MRI contrast agents based on MIONPs are being developed for highly selective detection of various types of cancer. Their optimal composition and functionalization are selected in terms of efficiency and toxicity. The optimal conditions for their use are also established.

The MIONS can accumulate in the tumor space due to increased blood flow, increased vascular permeability, and poor lymphatic drainage of tumors. This phenomenon has paved the way for the passive targeting of tumors and has been later termed the Enhanced Permeability (EPR) effect [26]. This effect is used to improve the quality of MRI tumor detection due to increased tissue density of the MNPs based contrast agent.

The Fe_3_O_4_-Au hybrid NPs were also proposed as MRI agents in [27]. The T_2_-weighted images were captured before, 30 min, 6 h and 24 h after NPs injection. Detection of octahedral-shaped Fe_3_O_4_-Au hybrid NPs accumulation was primarily observed in the liver 30 min after injection. Subsequently, at 6 h and 24 h following the injection, a noticeable improvement in tumor contrast was observed. The peak of NPs accumulation in malignant tissues was observed at roughly 6 h following the injection. Micelles loaded with MIONPs and coated by polyethylene glycol (PEG)-poly-caprolactone co-polymer were used as T_2_-weighted MRI contrast agents for brain tumors (glioblastoma multiform (GBM)) detection in a mouse model. The MRI-based visualization revealed an accumulation of the particles in both the heterotopic flank and orthotropic brain GBM tumors and provided reliable hypo intense MRI contrast enhancement with good delineation of tumor borders [28].

### 2.4. Targeted Contrast Agents

An effective approach for enhancing the accumulation of MNPs in tumors is to conjugate them with targeting segments [26]. This approach can amplify sensitivity by intensifying the density of MNPs in tissues, allowing for the development of tissue-specific contrast agents. This approach can also afford an opportunity to visualize the cellular and subcellular activities and mechanisms within living organisms non-invasively, promoting the trend also known as molecular MRI.

A novel type of T_2_-MRI contrast agent targeting the anti-human epidermal growth factor receptor 2 (HER2) built of MIONPs conjugated with HER2 antibody derivatives has been suggested [29]. The HER2 is closely associated with a negative prognosis and is overexpressed in different cancers ranging from ovarian, breast to gastric cancer, whereas its expression is considerably low in specific normal tissues. In vitro, human gastric cancer cells (N87) labeled by HER2-IONPs displayed negative contrast enhancement in T_2_-weighted MR images, in which the signal significantly decreased by 44.6 ± 7.8% than that in the control cells. In vivo, the percentage of signal decrease was 19.3 ± 5.3 and 8.4 ± 2.6%, for HER2-IONPs in the N87 tumor and IONPs covered PEG in the N87 tumor, respectively, thus increasing imaging sensitivity. The HER2-MIONPs successfully depicted HER2-positive tumors (human gastric cancer cells) using MRI.

The recombinant humanized monoclonal antibody Bevacizumab (BCZM) has been utilized to functionalize MIONPs [30]. The BCZM targets vascular endothelial growth factor-A (VEGF-A) to inhibit angiogenesis. This medication has been approved for the treatment of multiple types of metastatic cancers and is an optimal candidate for targeting tumor sites using VEGF-A targeting. The tumors appeared with high signal intensity on T_2_ weighted images taken 1 and 24 h after modified NPs injection. The Au coated Fe_3_O_4_ NPs were conjugated in the study [31] with epidermal growth factor receptor monoclonal antibody cetuximab (C225). These Au-Fe_3_O_4_-C225 hybrid NPs favorably targeted human glioma cell line U251 in vitro; they also possessed good targeting ability to xenografted glioma on nude mice in vivo and it showed excellent negative contrast media properties for MRI. Similarly, the prostate stem cell antigen antibody was bound to Au/MIONPs for diagnosis of prostate cancer [32].

The use of MIONPs in combination with epidermal growth factor receptor antibodies was explored [33] for detecting lung cancer via MRI. Results from an experiment conducted on C57BL/6 mice using the LLC1 cell line indicated that incorporating ligand antibodies led to greater MIONPs uptake in cancer cells, evidenced by an increase in signal intensity as measured via atomic absorption spectrophotometry. Thus, by tagging MIONPs with a ligand, it is possible to pinpoint cancer cells.

The MIONPs modified with short peptide sequences were proposed for the diagnosis of colon cancer [34] and fibro-sarcoma [35]. The mechanism of contrasting of such hybrid particles is based on the fact that peptide sequence recognizes integrin. Integrin receptors, especially α5β3, were found to be differentially overexpressed in tumors, playing a vital role in tumor angiogenesis [36].

Additionally, aptamers and peptides functionalization of NPs is used for liver cancer [37,38] visualization. The PEG coated MIONPs, and dextran (DEX)-coated IONPs conjugating folate on their surface are perspective molecular contrast agents for lungs cancer detection [39].

Polyethyleneimine-IONPs targeted by PNC27 peptide as a double targeting agent was proposed by [40] for early cancer diagnosis application.

### 2.5. Ultrasmall Superparamagnetic Iron Oxides Nanoparticles (USSPIONPs) as T_1_ and T_1_/T_2_ Contrasts Agents

Radiologists have a strong inclination towards T_1_-contrast agents because T_2_-contrast agents are known to create areas of darkness. Consequently, it is difficult to distinguish on T_2_ weighted images the affected tissues from air-tissue boundaries, internal bleeding, or other susceptibility artifacts. This in turn leads to a less precise diagnosis. Additionally, the high susceptibility of T_2_ contrast agents causes distortion of the magnetic field on neighboring normal tissues. This phenomenon known as a susceptibility artifact, or “blooming effect” creates indistinct images without any background over the lesions.

The USSPIONPs smaller than 5.0 nm in core diameter have gained more prominence as MRI contrast agents recently [41]. The decrease in size causes the USSPIONPs to have reduced magnetization due to the spin-canting effect, enabling them to efficiently decrease T_1_ relaxation time of water protons and make them appropriate for improving T_1_-weighted MRI. Additionally, their ultra-small size provides the USSPIONPs with the following benefits:

- By evading the nonspecific absorption by mononuclear phagocytes, these NPs can circulate within the body for an extended period, making them suitable for targeted imaging, steady-state imaging, and high-resolution imaging.
- Appropriate surface modifications enable these particles to clear through the kidneys, which reduces the risk of iron overload in patients with iron metabolism disorders, thereby improving biocompatibility and ensuring biosafety.
- Assembly/disassembly can be utilized to produce T_2_/T_1_ switchable contrast enhancement effects, thus improving the accuracy and sensitivity of MRI, making them a viable option.

Some USSIONPs with a size of approximately 3 nm and an ultra-thin hydrophilic shell of about 1 nm were produced by thermally decomposing Fe(oleate)_3_ in the presence of oleic acid. These particles were found to have a r_2_/r_1_ ratio of 2.0, which is lower than other SPIONPs-based positive contrast agents, but similar to Gd-based chelates [42]. Moreover, an in vivo MRI procedure was executed and exhibited that the contrast potency of the NPs was suitable for application in the given context. The optimized precipitation method produced protein-stabilized IONPs that were 2–3 nm in size, had favorable T_1_ and T_2_ relaxation times, excellent stability, and biocompatibility, indicating their potential for MRI usage [43]. Coated with poly-glucose, sorbitol and carboxymethyl USSPMIONPs were synthesized [44]. Average particle size ranged from 1.8 to 4 nm. The most successful T_1_ MRI contrast agent included NPs with average size of 3.7 nm. Its relaxivity r_1_ value was 4.11 (mmol L^−1^)^−1^ s^−1^ and an r_2_/r_1_ ratio during clinical 3 T MR scanning was of 7.90. Additionally, these NPs provided excellent T_1_ MRI contrast effects in water, cellular environments, and blood vessels.

### 2.6. Mixed Oxides as Contrasts Agents

Other nanosystems with varying magnetic cores have been developed to enhance MRI diagnostics and improve the signal sensitivity, in addition to NPs that rely solely on iron oxides for their metal core. Ferrites, which are mixed oxides resulting from substituting an iron oxide with a different metal ion such as Mn^2+^, Zn^2+^, Co^2+^, or Ni^2+^, are an example of such systems. The wet chemical co-precipitation method was used to prepare Co_1−x_Mn_x_Fe_2_O_4_ (x = 0.2, 0.4, 0.6, and 0.8) nano-ferrites that were coated with the biocompatible material, chitosan. The ferrites were tested for their ability to detect T_2_-weighted MR images, and it was found that the relaxation values decreased progressively with an increase in manganese ions [45]. The best contrast enhancement was observed with the contrast agent Co_0.8_Mn_0.2_Fe_2_O_4_. The Mn–Zn ferrites (Mn_0.6_Zn_0.4_Eu_x_Fe_2−x_O_4_, x = 0.00, 0.02, 0.04, 0.06, 0.08, 0.10, and 0.15) synthesized by the co-precipitation method and coated with citric or pluronic acids are suitable candidates for dual-mode MRI contrast agent positions [46]. An increase in the amount of europium to x = 0.15 results in a notable increase in the growth of r_1_ relaxivity. Conversely, substituting europium led to a decrease in r_2_ relaxivity as there was a decrease in saturation magnetization. The europium-free sample had an r_2_/r_1_ ratio of 152 which decreased to 11.2 for x = 0.15. Furthermore, manganese-zinc ferrite displayed a dual-contrast capacity, exhibiting higher longitudinal relaxivity (35.22 s^−1^ mM Fe^−1^) and transverse relaxivity (237.94 s^−1^ mM Fe^−1^) compared to Resovist^®^ [47]. The PEG-coated Mn, Zn ferrite NPs having a hierarchical structure and an average dimension of about 20 nm can be deemed as a promising T_2_ MRI contrast agent [48].

**Figure 1:**
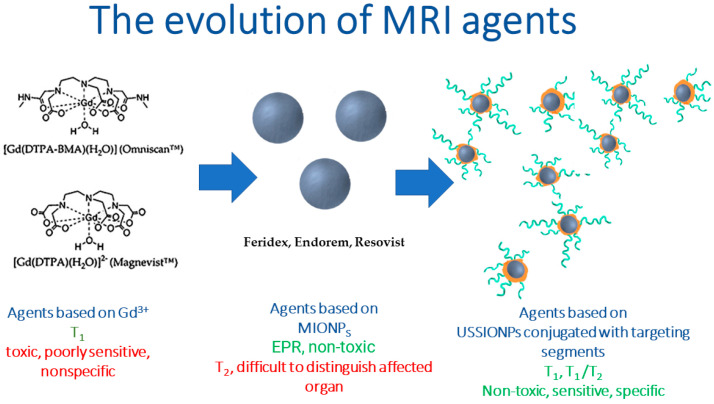
Past and future of MRI contrast agents.

The MRI agents discussed above are summarized in Table 1. This shows that over the past few decades, there has been an evolution of contrast agents. The changes are aimed at increasing the sensitivity and contrast of the method and reducing the toxic effect. New, less toxic than Gd-chelates T_2_ contrast agents based on MIONPs have appeared. Further development of the method is associated with the creation and application of target agents that find and allow the sensitive detection of tumor cells due to targeted accumulation. Probably, these studies will be carried out using USSPMIONPs or ferrites NPs due to their potential as T_1_ and T_1_/T_2_ MRI contrast agents (Figure 1). Research is also ongoing to improve the effectiveness and biocompatibility of contrast agents by modifying their surface with various coatings and vectors, changing their magnetic properties by controlling their composition.

## 3. Applications of MNPs for Cancer Heating Therapy

Hyperthermia (HT) is a type of treatment in which body tissue is exposed to high temperatures to damage and kill cancer cells or to make cancer cells more sensitive to the effects of radiation and certain anticancer chemotherapeutic drugs. The term “hyperthermia” is derived from two Greek words, “hyper” and “therme,” meaning “rise” and “heat,” respectively [49]. The treatment is based on the observation that tumor cells can be destroyed by heating the cells for some time to temperatures between 43 and 46 °C while healthy cells are less affected. Cancer cells’ susceptibility to high temperatures stems from a lack of oxygen within them due to an inadequate blood supply in the affected area. By exposing these cells to temperatures ranging between 41 °C and 46 °C for a minimum of 20–60 min, their proliferation can be curtailed [50,51]. In the cases of temperature above 46 °C, treatments lasting even just a few minutes can lead to the death of cancer cells. However, this is offset by the harm caused to viable cells, leading to occurrences like coagulation, carbonization, or tissue necrosis.

### 3.1. HT Types

HT can be divided into several types as follows:

- Whole body (WB) HT.
- HT by wireless applicators (WA).
- HT by heating source insertion (HIS).
- Magnetic hyperthermia (MHT).

Whole body HT, carried out with the help of hot water bath, hot water blankets, thermal chambers, is the primary method of hyperthermia. It does not allow heating selectivity and is a commonly used metastatic cancer treatment. The WA HT is already a local or regional method. It is associated with the use of high-intensity focused ultrasound or electromagnetic applicators (radio frequency and microwave) for heating. The disadvantages of the method are the uneven distribution of heat, its insufficient penetration into the affected area and the heating of unaffected tissues. Of all the possible implementations of the method, microwave WA HT is the most efficient; however, even in this case, there is no uniformity of temperature around deep-seated tumors. Inserting radio frequency or microwave heating sources inside or around the affected area through surgery (HIS) is recommended to reduce heterogeneity of heat distribution in cells. However, this invasive method causes complications and discomfort for patients during and after therapy.

**Figure 2:**
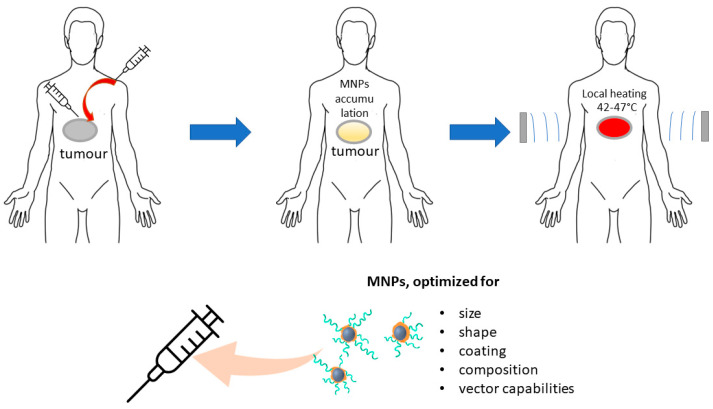
MHT method scheme.

Hence, the HT techniques mentioned above exhibit substantial shortcomings in terms of targeting tumors and accurately directing thermal energy. The optimal approach for HT should be minimally invasive, targeted toward specific tissues, and capable of delivering high-intensity heating in deeper tissues with precision. The MHT, a unique form of HT facilitated by nanotechnology, offers a revolutionary solution that overcomes the drawbacks and diminishes adverse effects associated with other HT methods. The application of MHT facilitates the production of local heat at a distant location by utilizing magnetic energy losses of MNPs (which may have been actively or passively accumulated in the tumor), under the influence of an oscillating magnetic field (Figure 2). Thus, by converting electromagnetic energy into heat, certain MNPs can induce temperature elevation in specific areas of the human body where tumor cells and NPs are present. Hence, regulating the activation of these NPs by means of an oscillating magnetic field can allow for external control of them as nanoheaters.

### 3.2. Types of MNPs Suitable for HT and Their Requirements

The MNPs which have been studied for use as HT agents comprise two categories based on their structure: magnetic metal or alloy NPs and magnetic metal oxide NPs.

The metals commonly utilized for HT applications are nickel (Ni), iron (Fe), and cobalt (Co) due to their magnetic features, including high saturation magnetization, large magnetic anisotropy, and high magnetic moment. Nevertheless, these metallic magnetic NPs have challenges that include inadequate chemical stability and biocompatibility, susceptibility to oxidation, and pyrophoricity at room temperature. These downsides lead to partial or complete loss of magnetization that renders them unfit for HT use. However, it is possible to improve the resistance of the magnetic metal component towards oxidation by incorporating additional metals into NP compositions or using alternative covers such as polymers, organic materials, or ceramics. Furthermore, NPs of magnetic metal alloys were used to optimize the magnetic properties. For the MHT needs, as magnetic agents Fe [52], Co [53] NPs and Fe-Ni-Co [54], Co-Ni [55], Fe-Au [56,57,58], Fe-Al [59], Fe-Rh [60], Fe-Cr-Nb-B [61], Cu-Fe and Cu-Ni [62] alloys are considered. The potential chemical instability and toxicity of Co and Ni limit their use.

Metal oxide MNPs are highly stable when exposed to oxidation, making them frequently employed for MHT purposes. Of all the metal oxide MNPs, MIONPs are the most extensively researched due to their exceptional self-heating properties. The iron oxide forms of magnetite and maghemite are also the most desirable options for MHT due to their chemical stability, diminished toxicity, and biodegradability [63,64,65,66].

The replacement of ferrous iron in magnetite and the formation of a mixed oxide makes it possible to change the properties of MNPs, for example, magnetic (Co, Ni) or antibacterial (Zn, Cu). The MHT agents can be ferrites such as Co-Fe, Ni-Fe [67], and Ca-Mn [68], Zn-Mn [69], Cu-Co [70], Co-Mn [45] mixed ferrites.

For MNPs to effectively work in MHT, various factors such as targeting, clearance, and heating efficiency need to be taken into consideration. The heating efficiency is calculated by assessing the specific absorption rate (SAR) which represents the MNPs’ ability to eliminate cancer cells. The SAR value of the sample can be calculated by means the equation:

$$SAR=C_{p,s}\frac{dT}{dt}$$

where C_p_,_s_ is the heat capacity of the imaging tissue, dT/dt is the initial slope of the time-dependent heating curve. The heating mechanisms that contribute to SAR are attributed to hysteresis loss in multi-domain magnetic particles that are larger than 1 μm, relaxation loss in single-domain SPMNPs and eddy currents in magnetic particles that are larger than 1 μm. Several factors, which include saturation magnetization, size and shape of MNP particles, T_H_, intensity, and frequency of the alternating current magnetic field can influence the SAR value.

### 3.3. Effect of Size and Shape on MHT Properties

To ensure effective heating of the NPs when subjected to an oscillating magnetic field, it is important to aim for as high a saturation magnetization as possible. The MNPs exhibit superparamagnetic traits, which are dependent on the size of the particles. By increasing the size of the particles, the magnetization saturation values also increase, which ultimately translates to improved MHT application performance. However, there is a critical size threshold, also referred to as the superparamagnetic limit, that, when surpassed, renders NPs magnetic. Additionally, the influence of particle size on the possibility of their delivery, including the possibility of overcoming biological barriers, must also be considered. The correlation between the size (varied from 5 nm to 110 nm) of silica-coated Fe_3_O_4_ MNPs and the SAR value has been investigated [71]. The SAR values ranged between 137 and 1 W/g for particle sizes of 24 and 110 nm, respectively. With a shift from mono-disperse to poly-disperse MNPs, there is a decline in the SAR value due to the decrease in particle size uniformity, resulting in increased total heat generation. Consequently, the size distribution significantly affects the induction heat value. Increasing the particle size of cubic cobalt ferrite NPs from 20 to 27 nm leads to saturation magnetization growth from the value of 50 emu/g to the value of 62 emu/g. Substantial reduction in SAR values was observed after eliminating the alternating current magnetic field, which suggests a decrease in the heat energy that damages cancer cells while preserving magnetization [72]. Maghemite NPs in the size range of 5–16.5 nm have shown a maximum SAR of 1650 W/g for NPs with a size of 16.5 nm [73]. The MIONPs of different sizes ranging from 5 nm to 110 nm were utilized by Gonzalez-Fernandez et al. [71] to determine the maximum heating efficiency. Their experiments indicated that the most effective size was just below 30 nm. In a separate study, Dr. Wang examined magnetite NPs with diameters of 6 nm, 8 nm, and 10 nm and determined that the 10 nm particle exhibited the greatest efficiency [74]. Furthermore, Gonzalez-Weimuller tested USSPIONPs ranging between 5 nm and 14 nm using a field strength of 24.5 kA/m and a frequency of 400 kHz and found that the optimal size was 14 nm.

The particle size dependence of SAR had a maximum at a particle size of just about or slightly less than one magnetic domain. The use of bulk particles in MHT treatment is limited if they possess a complex domain structure as magnetization reversal occurs when the magnetic moments flip in domains that are antiparallel to alternating magnetic fields. Additionally, magnetic domain growth occurs in other domains at lower field strengths [75]. The single-domain MNPs experience significantly greater power loss than the multi-domain NPs. Therefore, if the magnetic material is fully saturated, an increase in domain size should result in a decrease in power loss. Consequently, to reduce power loss, it is recommended to avoid larger domain particles [74].

For biomedical applications, single-domain superparamagnetic NPs (SPNPs) are preferred over multidomain ferri- and ferromagnetic NPs. The use of SPNPs means that the magnetization drops to zero when the applied magnetic field is removed.

The alteration of shape has been identified as a promising approach for improving the magnetic properties by introducing additional anisotropies and consequently increasing the heating efficiency. It is possible to adjust the shape anisotropy of MNPs with diverse morphological features to enhance their performance. Among FeO/Fe_3_O_4_ NPs, cube shaped MNPs with superior surface anisotropy exhibited a higher SAR of 200 W/g compared to spherical MNPs with a SAR of 135 W/g [76]. When compared to spheres (140 W/g) and cubes (314 W/g) of equivalent volumes, iron oxide nanorods display a greater SAR value of 862 W/g [77].

The amount of induced heat power is directly affected by changes in frequency and amplitude of the alternating magnetic field. Typically, SAR values rises with an increase in frequency, but caution must be taken within a restricted range.

### 3.4. Effect of Coating on HT Properties

A variety of coatings are employed for MNPs to accomplish diverse tasks such as:

- Preserving MNPs’ physiochemical properties and composition.
- Enhancing MNPs’ biocompatibility while also reducing toxicity since their surface comes into direct contact with blood and tissues.
- Inserting hydrophilic molecules on the surface to enhance the dispersity of SPMNPs, which in turn prevents agglomeration, controls particle size, reduces the possibility of blood capillary obstruction, and improves blood circulation by transporting SPMNPs to targeted areas.
- Altering the surface to create a more suitable platform for further functionalization and protein absorption.
- Preventing the SPMNPs opsonization.

When creating new magnetic agents for HT, it is important to take into account the potential impact of the coating on the magnetic properties of MNPs.

A broad range of coatings can be utilized for MNPs, which can be classified into two primary groups: organic and inorganic, each with subcategories. The organic category encompasses surfactants, polymers, and biological molecules, while the inorganic group comprises metals/non-metals, metal oxides/sulfides, and silica. The magnetization of spherical Fe_3_O_4_ NPs in the range of 4–11 nm in diameter was investigated for in vitro applications [78], with the aim of determining the impact of different polymer coatings including PEG, DEX, polyvinylpyrrolidone (PVP), and bovine serum albumin (BSA). The saturation magnetization of Fe_3_O_4_ without any coating was recorded as 64.35 emu/g and exhibited a noticeable decrease after modification. The values were recorded as 58.42 emu/g for PEG, 56.59 emu/g for DEX, 55.70 emu/g for PVP, and 58.64 emu/g for BSA. The Fe_3_O_4_@Ag variant demonstrating superior biocompatibility exhibited a saturation magnetization value of 75.1 emu/g and SAR values of 76 W/g [79]. This suggests that the MHT efficiency was not significantly diminished by the thin layer of Ag coating.

### 3.5. Targeting Cancer Cells with MNPs for MHT

Similarly, to how magnetic agents are used in MRI, magnetic agents infused via injection for MHT that are based on MNP may gather in the tumor zone owing to the EPR effect [80]. The magnetic qualities of MNPs enable their distribution to be externally controlled by utilizing an external magnetic field to steer them towards the targeted zone [81]. Functionalizing the surface of MNPs with agents that interact with specific receptors overexpressed by the target cells is the most reliable and commonly used method for delivering MNPs. In the case of tumor cells, the same targeting agents used for MRI are employed.

The utilization of antibody-bound MIONPs hinted at the potential to create a targeted thermal treatment for metastatic cancer by means of induction heating with an external alternating magnetic field. Tests were conducted employing (111) In-ChL6 bio-probes (a chimeric L6) in an artificial xenograft model for human breast cancer [82]. Another [83] examination administered Herceptin (trastuzumab) to transport ferric oxide-enriched NPs to Human Epidermal Growth Factor Receptor 2 (HER-2+) cancer cells. The effectiveness of the application of alternating magnetic field activated Herceptin-directed NPs was validated in the selective elimination of HER-2+ human cancer cells.

The MHT is a more effective and harm-reducing replacement for WBHT, WAHT, and HIS HT. The use of MNPs with a size less or equal one magnetic domain, as well as taking into the account the influence of the composition, shape of particles and their coatings on SAR, is the next important step to further increase the potential of MHT (Figure 2). Conjugation of the NPs with specific vector segments is also a valuable step in the further modification of magnetic agents.

## 4. Photo-Thermal Therapy and Photo-Dynamic Therapy (PTT and PDT)

### 4.1. PTT

The optical properties of NPs are utilized in photo-thermal therapy (PTT), which is a platform to fight cancer by using multiplexed interactive plasmonic nanomaterials as probes in combination with the excellent therapeutic performance of near-infrared (NIR) light. The NPs that have SPR could convert light into heat. Induced localized temperature increase leads to a hyperthermic response in the targeted tissue. The light absorption wavelength can be tuned by altering the shape and size of the NPs.

The technique boasts several advantages [84], which include:

- Precise targeting of therapy to particular tissues to prevent unintended effects due to high radiation absorption caused by the resonance wavelength of NPs.
- The ability of NPs to reach tumors located in deeper tissues through attachment of specific agents to the surface of Au NPs that augment their specificity for selected tumor cells.
- Possibility of merging NPs PTT with drug treatment as NPs serve as carriers for drugs to designated tissue locations, thereby amplifying their curative potential.

For PTT, the criteria used to select NPs are based on specific traits [85]: the ability to absorb in the NIR range (700–1000 nm), size smaller than 100 nm to maximize tissue absorption, enhanced absorption cross-section, decreased toxicity, and improved biocompatibility. Gold is considered as one of the most appropriate and thoroughly investigated plasmonic material due to its minimal harmful effects on cells [86,87,88], absorption in the near-infrared spectrum [89], proficiency in transforming light into heat energy [90], and its availability in diverse morphologies [91].

The study [92] produced mesoporous Au-Pt NPs that were labeled with a cell-targeting ligand (folic acid), a mitochondria-targeting group (triphenylphosphine), and a photosensitizer. These NPs can serve as a phototherapeutic agent with dual mode capabilities for enhanced cancer therapy and molecular targeting of disease progression. The nanoparticles’ ability to convert laser radiation into heat can result in thermally induced cell damage. Furthermore, the systems have demonstrated a noteworthy enhancement in the efficacy of photodynamic therapy (PDT) with improved cellular uptake, effective generation of mitochondrial ROS burst, and intelligent release of oxygen. Magnetic Fe_3_O_4_-Au NPs have been utilized to combine radiotherapy (RT) and photothermal therapy (PTT) [93]. The hybrid NPs possess exceptional surface plasmonic resonance characteristics, impressive superparamagnetic properties, high photo-thermal conversion efficacy, and good biocompatibility. When subjected to near-infrared radiation for the brief period, cervical cancer cells in vitro perish with a low concentration of Fe_3_O_4_-Au NPs. The application of RT and PTT have demonstrated in vitro synergistic anti-cancer effects. The Fe_3_O_4_-SiO_2_-Au NPs were developed [94] for treating cancer cells using MRI-guided chemo/NIR photo-thermal therapy. The study employed two colon cancer cell lines namely SW480 and SW620 along with a laser having a wavelength of 808 nm and a power density of 100 mW cm^−2^. The Fe_3_O_4_-SiO_2_-Au NPs were found to be minimally toxic to cancer cells at around 10–15%. However, upon exposure to laser irradiation, the mortality rate of the cells increased drastically, leading to approximately 43–50% cell death.

### 4.2. PDT

Photo-dynamic therapy is a two-step treatment that uses a photosensitizer drug to eliminate cancerous and precancerous cells after the activation by the light. The photosensitizer is activated by a laser, typically emitting a specific wavelength of light energy. To carry out PDT, the first step is to inject a photosensitizer into the specific tissue that is to be treated. The next step is to apply a certain wavelength of light that triggers the photosensitizer to produce reactive oxygen species (ROS) through energy transfer, thereby leading to cellular apoptosis. Gold containing NPs are used not only as PTT agents, but also as PDT photosensitizer or photosensitizer carrier.

A hybrid system of Au-NPs and methylene blue, a well-known photosensitizer, was created by utilizing an intermolecular interaction between a polystyrene-alt-maleic acid (PSMA) layer on the Au-NPs and methylene blue. The resulting Au-PSMA-methylene blue hybrid NPs exhibited a high quantum yield of singlet oxygen molecules, exceeding 50% of that of free methylene blue, when excited by dark red-light at 660 nm, without causing any significant dark toxicity. To improve selectivity towards cervical cancer tumor (HeLa) cells in comparison to mouse embryonic fibroblasts (3T3 fibroblasts), the hybrid NPs were conjugated with transferrin. By subjecting the Au-PSMA- methylene blue—transferrin NPs to a single hand-held laser treatment (32 mW/cm) lasting for 4 min, the PDT efficiency doubled [95].

To achieve synergistic drug therapy and PDT, Akram, M. W. developed Au-TiO_2_ NPs bound with doxorubicin [96]. These hybrid systems, comprising Au-TiO_2_ and doxorubicin, can easily infiltrate the vicinity of the tumor due to the acidic nature of core tissue. When exposed to an optimal amount of ultraviolet light, Au-TiO_2_ stimulates doxorubicin to act as a photosensitizing agent. The production of significant ROS by pure Au-TiO_2_ has the ability to eliminate around 70% of cancerous cells (specifically breast malignancy cells). However, when subjected to laser irradiation, the percentage rises to 82% for Au-TiO_2_-doxorubicin. Based on the findings, the combination of doxorubicin with Au-TiO_2_ shows greater potential and efficiency compared to using only pure Au-TiO_2_.

Additionally, Au–Bi bimetallic nanoparticles, which have a size of 5 nm, have been found to be effective agents for near infrared light-induced phototherapy for cancer treatment [97]. This is due to their ability to generate ^1^O_2_ while exhibiting exceptional photo-thermal conversion efficiency (η = 34.2%) under 808 nm laser irradiation. Other promising candidates for photosensitizers include Au-CoFe_2_O_4_ nanostructures with a spiky gold layer [98], silica-Au-PEG-doxorubicin [99], and Pd-tipped Au nanorods [100]. For PTT and PDT application, 4-carboxyphenyl porphyrin-conjugated silica-coated gold nanorods were synthesized in [101].

The application of NPs and nanotechnologies is also linked to the development and execution of cancer therapies like PDT and PTT. However, also as for MHT, these treatments still have ample opportunities for refinement. Altering the size, shape, coating, and composition of nanoparticles, as well as the characteristics of the vectors, can decrease toxicity and improve the efficiency of these treatments.

## 5. Applications of INPs for Drug Delivery

### 5.1. NPs Based Delivery Systems

The main disadvantage of most chemotherapeutic agents is their relative non-specificity and therefore potential side effects on healthy tissues. Therefore, in the field of pharmacology, special attention is paid to the delivery of drugs and the control of the release of therapeutic substances in localized targets, especially in the field of cancer treatment.

The NPs of metals and their oxides are among the most promising drug carriers, due to their biocompatibility and diverse drug loading possibilities. They can not only be easily loaded with a medicinal substance, but also functionalized with target agents, and provide localization of drug nanosystems to a diseased tissue. Of particular value as carriers of medicinal systems are ferro-, ferri- and superpara- magnetic NPs. Due to their magnetic properties, they can increase the effectiveness and reduce the systemic toxicity of drug systems.

The primary benefits of MNPs include their ability to be:

- Visualized (utilizing ferro-, ferri-, and superparamagnetic NPs for MRI).
- Controlled or fixed in position via a magnetic field.
- Subjected to a magnetic field for heat-induced drug release or tissue hyperthermia/ablation.

In general, this targeted delivery process involves attaching a cytotoxic drug to biocompatible carriers—NPs or MNPs, introducing these hybrid systems into the body, or localizing to a pathological site, and releasing a therapeutic agent. While this seems simple, there are many variables that make this technique difficult to perform. It is necessary to consider such parameters as the physicochemical properties of NPs-based hybrid systems, their biocompatibility, toxicity, interaction with the body’s immune system, the depth of the target tissue, the optimal method of targeting tumor or virus cells, blood flow velocity and vascular supply, the optimization of the time and place of drug molecules’ release and more.

**Figure 3:**
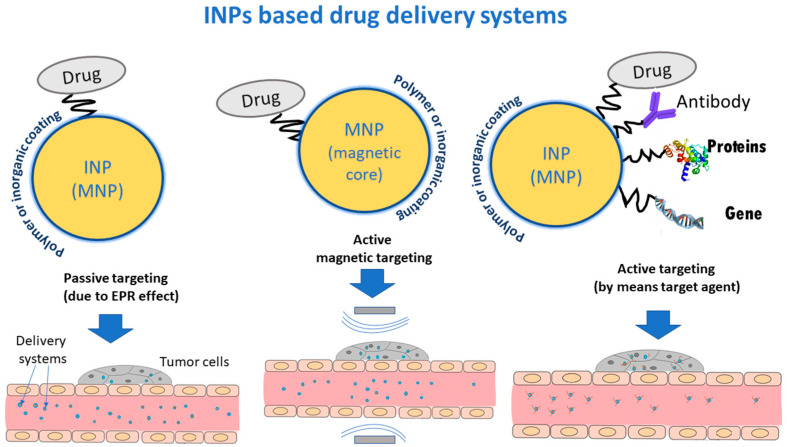
Drug delivery systems based on INPs and their targeting strategies.

Therefore, a drug delivery system should include INPs (as carriers), a medicinal component and agents that provide targeting the affected organ. Coatings or linkers are also needed to link the various parts of the hybrid system. Coatings can also act as stabilizer, improve biocompatibility, and reduce toxicity in hybrid systems (Figure 3). In a case when the INPs are also MNPs they can perform not only the function of a carrier, but also be a magnetic vector, an agent for imaging (MRI) or MHT.

### 5.2. Delivery Strategies for Drug Systems

#### 5.2.1. Passive Targeting

For drug delivery, nanocarriers could be accumulated in the tumor by passive targeting via EPR. Passive drug targeting leverages the biophysical features of tumors to enhance the extravasation of NPs into the tumor microenvironment [102]. As per the EPR effect, the shape, size, and surface charge of the drug largely govern the drug transport in cancer [103]. However, due to the heterogeneous nature of solid tumors, the EPR-mediated passive drug targeting leads to non-uniform distribution of the drug, thereby increasing the chances of local cancer recurrence [104].

#### 5.2.2. Magnetic Targeting

The problem of non-specificity of most chemotherapeutic agents can be solved by means of magnetic drug targeting [105]. This strategy is based on the attraction of magnetic nanocarriers to an external magnetic field.

The procedure consists of the following stages:

- Fastening a biocompatible MNPs carrier to a cytotoxic drug.
- Infusing these hybrid nanosystems as a colloidal suspension through an intravenous injection.
- Employing a magnetic field gradient to steer these hybrid nanosystems towards the affected area.
- Directed release the medical substance from the drug system.

The effectiveness of magnetic drug delivery is affected by the physicochemical characteristics and composition of the carriers involved. Improved magnetization plays a critical role in simplifying the administration of medication. Superparamagnetic NPs are favored because they are magnetized by an external magnetic field and readily lose their magnetism once the field is no longer applied. Magnetic iron oxides are more often considered as magnetic carriers due to their low toxicity.

Research conducted on a rabbit model demonstrated [106] that tumors can be effectively infused with the chemotherapeutic drug mitoxantrone using a magnetic carrier system comprised of MIONPs (hydrodynamic particles with a diameter under 200 nm). Magnetic targeting experiments revealed that the highest drug accumulation (57.2%) was observed in the tumor region, while lower accumulations were found in the liver (14.4%) and kidneys (15.2%) relative to the recovery from all examined tissues. Passive targeting yielded different results, with the tumor region accounting for 0.7%, liver for 14.4%, and kidneys for 77.8%. Through magnetic targeting and utilizing only 5–10% of the typical chemotherapy dosage, the treatment led to full tumor remission. In a single dose of these minimal amounts, complete tumor remission was observed in approximately 25% of the animals treated, with no significant local or systemic side effects.

Despite that colloidal MIONPs loaded with epirubicin and targeted towards solid tumors were shown to exhibit accumulation in the intended target more than two decades ago [107], magnetic targeted delivery systems have not yet reached the pharmaceutical’s market due to the numerous issues that have arisen. The extent to which the magnetic gradient weakens as the target’s distance increases is the primary constraint of magnetic drug delivery. This constraint is closely linked to the maximum strength of the external field that could be administered to acquire the required magnetic gradient, which determines the duration of NPs’ habitation within the intended space or initiates medication release. To align with the International Commission of Non-ionizing Radiation Protection’s recommendation [108] for clinical employment, patients must not receive magnetic flux densities exceeding 400 mT in any bodily region. Magnetic drug delivery has been utilized in preclinical research magnetic fields with intensities ranging between 0.1 T and 1.5 T [109]. Delivery effectiveness is significantly impacted by both the distance between the magnet and the targeted delivery site, as well as the magnet’s geometry. Effective magnetic field depths of up to 5–15 cm in the body can be achieved by utilizing Permanent Nd-Fe-B magnets along with SPION, which exhibit outstanding magnetic properties [109,110]. However, delivering therapeutic agents to deep tissues in vivo remains a significant challenge in magnetic drug delivery because MNPs can be efficiently controlled in the superficial tissue layers while the target regions remain unreachable.

A further restriction concerns the size of MNPs, which must be small to achieve superparamagnetic behavior and prevent magnetic clumping when the magnetic field is withdrawn. However, this small size results in a weaker magnetic response that makes it challenging to manipulate the particles and maintain their proximity to the target while resisting the force of blood flow [111]. Moreover, magnetic delivery should consider many factors, such as toxicity and biocompatibility of magnetic carriers, blood flow velocity and vascular supply, timeliness of drug release.

#### 5.2.3. Active Targeting

As mentioned earlier, MNPs can be attached to target agents to enhance the targeted delivery of NPs in diseased tissue. Ligands typically employed for targeting tumor cells include small molecules (e.g., proteins (such as asialoglycoprotein receptor), folic acid for cancer [111,112]), peptides (such as R-Tf-D-LP4, voltage-dependent anion channel 1 (VDAC1) based peptide), and nanoantibodies [113,114].

The strategies discussed above find applications in the creation of both antitumor and antiviral targeted delivery drugs.

## 6. Applications of Metal and Metal Oxides NPs for Delivery of Anticancer Agents

### 6.1. Targeted Delivery Systems of Different Anticancer Agents Based on MNP_S_

#### 6.1.1. Conventional Chemotherapeutic Agents

Magnetic nanoparticles have been evaluated as drug carriers for a variety of chemotherapeutic agents, first of all traditional drugs. Carriers can be designed with specific characteristics to enhance the efficacy of these therapeutic agents over that achieved by typical systemic delivery. Characteristics such as loading capacity and drug release profiles can now be tailored by controlling the structural features and chemical bonding in the MNP conjugate.

Yang et al. utilized magnetite NPs coated with poly(ethyl-2-cyanoacrylate) (PECA) to incorporate the anticancer agents cisplatin and gemcitabine. The release of cisplatin was more gradual due to its hydrophobic nature, while the hydrophilic gemcitabine had a more rapid release [115]. Alternatively, Kheiri loaded the anticancer drug 5-fluorouracil (5-FLU) into Fe_3_O_4_@ chitosan-polyacrylic acid nanogel particles with a chitosan-polyacrylic acid shell [116]. Drug release tests were performed in two different conditions; simulated physiological environment (pH 7.4) and tumor tissue conditions (pH 4.5) to evaluate the release behavior of nanoparticles. The results showed an elevated release rate of 5-FLU from the core-shell NPs in tumor tissue conditions.

Smart magnetic nanocarriers containing doxorubicin were created using flower-shaped magnetite NPs with average size 16.4 nm, which were enclosed within a poly (N-vinyl caprolactam-co-acrylic acid) copolymer that is responsive to changes in temperature and pH-value. The system was successful in controlling drug release to a high degree [117], as proven by its ability to achieve encapsulation efficiency of over 96% when loaded with doxorubicin at neutral pH-value. An almost complete release of the drug was seen in acidic pH during high-temperature conditions, whereas a minimal amount of doxorubicin was let out in neutral pH at body temperature. The doxorubicin anticancer drug was loaded into nanocarriers containing surface-altered MIONPs with crosslinked Pluronic F127 and branched poly-ethylenimine [118]. At a pH of 5.4, a high rate of 54.8% doxorubicin release was observed within 48 h, and at 42 °C (pH 7.4), a release rate of 51.0% was observed. Obtained delivery systems indicated a high cellular uptake enhanced by alternating magnetic field. The Fe_3_O_4_ MNPs coated with silica and covalently modified with [(3-triethoxysilyl)-propyl]-succinic acid–polyethylene glycol exhibited high levels of doxorubicin loading. The study assessed the effectiveness of removing drugs from nanomaterial surfaces using an alternating magnetic field in both acidic and neutral environments. It was found that the optimal pH for efficiently removing doxorubicin was 5.8 [119].

The Fe_3_O_4_–oxaliplatin @ chitosan core–shell and Fe_3_O_4_–irinotecan @ chitosan core–shell hybrid nanosystems are proposed for colorectal cancer cells therapy [120]. Magnetic poly-(D, L-lactide-co-glycolic acid) (PLGA) microspheres loaded with an anticancer drug 5-FLU were prepared [121] via solvent evaporation from a water-in-oil-in-water ternary emulsion system. The magnetic nanoparticulate system exhibited pH-dependent release of 5-FLU.

Testing of new drug systems takes place not only in vitro but also on animal models. Multilayered polymer microcapsules loaded with magnetite and doxorubicin and modified with designed Ankyrin repeat protein were proposed [122] for targeted lung cancer therapy. In vivo and ex vivo bio-distribution studies in mouse models showed that the carrier surface modification with the protein changed the bio-distribution of the capsules toward epithelial cells. In particular, the capsules accumulated substantially in the lungs. Magnetic silica nanocomposites decorated with Pluronic F127 and loaded with doxorubicin were tested for hepatocellular carcinoma therapy. The hybrid composites exhibited a significant therapeutic effect against human liver cancer (HepG2) cells. Mice tumors treated with the composites exhibited the highest level of cell necrosis with membrane and vacuole destruction compared to free doxorubicin. Mice treated with magnetic nanosystems without drug molecules possessed tumors with large regions of viable cancerous cells and immense vascular structures [123].

Previously covered examples of anticancer prodrug candidates are shown in Table 2.

#### 6.1.2. Genes

Due to its capacity to revolutionize medicine, gene therapy has been a subject of intense inquiry in recent years. For cancer gene therapy to be effective, a smart gene delivery system is needed for both protecting therapeutic genes from degradation in circulation and enabling robust gene expression at the tumor sites. The potential for effective delivery of genes to tumor tissues, compatibility with biological systems, magnetic response, and adaptability through surface modification make MNPs a promising platform for gene-delivery systems. This approach involves loading IONPs with negatively charged genetic drugs through electrostatic adsorption [124].

A local chemotherapy agent relying on IONPs was introduced by Zhang et al. to manage glioblastoma in postoperative patients, targeting ferroptosis and apoptosis. The porous configuration of IONPs with attached carboxyl groups was used as a carrier for codelivery of cisplatin and small interfering ribonucleic acid (siRNA) targeting glutathione peroxidase 4 (GPX4) and exhibited significant drug loading efficiencies. The hybrid systems that were created had a significant impact on glioblastoma cells in U87MG and P3#GBM, whereas their impact on normal human astrocytes was minimal. The mechanism by which the tumor cells were affected was complex. The ROS were produced through the interaction of Fe^2+^ and intracellular H_2_O_2_, leading to ferroptosis initiation. Additionally, the co-released si-RNA suppressed GPX4 expression, while cisplatin caused damage to nuclear deoxyribonucleic acid (DNA) and mitochondrial DNA, thereby inducing apoptosis [125].

One more illustration of the capability and success of utilizing IONPs for simultaneous administration of gene drugs and chemotherapeutic drugs can be found in [126]. The MIONPs coated with polyethyleneimine and polyethylene were employed to transport both microRNA-21 antisense oligonucleotide and gemcitabine to pancreatic cancer cells. This concurrent delivery approach efficiently suppressed the proliferation and spreading of cancerous cells by hindering the epithelial-mesenchymal transition and enhancing the activity of tumor suppressor genes PTEN and PDCD4.

The application of MNPs with curcumin-coated chitosan and MNPs with pCEM-TRAIL plasmid-coated chitosan was studied in inducing apoptosis in B16F10 tumor cells. This was done either alone or in combination with the aim of overexpressing the receptors required for apoptosis induction. Results revealed a significant increase in cellular death within 48 h when using a combination of TRAIL gene-based and drug-based systems compared to each nanocomplex individually, indicating a synergistic effect [127].

The introduction of exogenous gene-drug-carrying IONPs into the body can trigger the immune response. As a result of this immune activation, a synergistic response may be induced, allowing for the identification and elimination of tumors [128].

#### 6.1.3. Proteins and Peptides

Magnetic nanoparticles have been explored not only as transporters of drug molecules but also for therapeutic proteins and peptides. Tumor-penetrating peptide (iRGD) coated IONPs showed effectiveness against brain metastasis of breast cancer supported by the tumor-penetrating nature of this peptide. Tumor progression suppression was observed in a mouse model by administering medical systems intravenously. The study [129] showed that administering a single dose of the preparation early in the tumor development stage can have a noteworthy impact on the progression of metastatic tumors and the retention of nonproliferative cancer cells. Magnetic nanoparticles conjugated with Herceptin™ (trastuzumab) act as a monoclonal antibody targeting agent [130]; however, they also have a therapeutic effect in reducing cell proliferation by inducing cell arrest during the G1 phase of the cell cycle [131].

### 6.2. Targeted Delivery Systems of Different Anticancer Agents Based on Non-Magnetic NP_S_

Non-magnetic INPs are also utilized for both passive and active targeting of tumor tissue [86,132]. Silver NPs with a diameter of 30–40 nm, capped with epirubicin, were synthesized using a one-pot method in which epirubicin served as both the reducing agent and functional drug. These NPs exhibited an IC_50_ (half maximal inhibitory concentration) of 1.92 μg/mL against Hep G2 cells [133]. Spherical-shaped Ag NPs of 6 nm size were produced using *Aerva javanica* extracts and loaded with the anti-cancer drug gefitinib. According to research, the use of Ag NPs in combination with gefitinib results in a reduction of over 50% MCF-7 cell viability and increased apoptosis in cancer cells, compared to the use of gefitinib alone [134]. Similar benefits have been observed in other cases where drugs were incorporated into compositions with Ag NPs. Thus, alendronate showed limited effectiveness in inhibiting cancer cell growth, with an inhibition maximum of 47% at a concentration of 500 µM. The hybrid systems of Ag NPs and alendronate exhibited remarkable potency with an IC_50_ of 10.1 µM, possibly owing to their elevated lipophilicity and enhanced cellular uptake in comparison to free alendronate [135]. In addition, Ag NPs that were coated with PEG and contained methotrexate, exhibited stronger anti-cancer properties towards MCF-7 cells, with an IC_50_ of 258.6 µg/mL, whereas the drug alone had IC_50_ of 512.7 µg/mL. Probably, the increased antitumor activity of combinations of Ag NPs and antitumor drugs is because silver NPs themselves exhibit antitumor activity [136].

Due to their desirable qualities of size, stability in various conditions, hydrophilicity, and biocompatibility, Au NPs have gained attention as potential drug-delivery agents. Bovine serum albumin was used as both a reducing and capping agent to create gold NPs with an average diameter of 50 nm, which were then used to deliver the anticancer drug methotrexate. The study found that the drug was released faster at pH 4 than at pH 7.4. The nanosystems that were acquired demonstrated greater cytotoxic effects on MCF-7 cells than an equivalent amount of free methotrexate. This heightened effectiveness is believed to be the result of the MCF-7 cells’ preferential absorption of the hybrid particles, due to the presence of BSA that supplies nutrients and energy to the cells that are rapidly proliferating. Additionally, the methotrexate drug’s ability to target the over-expressed folate receptors on MCF-7 cells contributes to the improved absorption and effectiveness of the drug [137].

To conduct loading and release experiments on two promising copper(I)-based antitumor compounds, [Cu(PTA)_4_] + [BF4] (compound A; PTA stands for 1,3,5-triaza-7-phosphadamantane) and [HB(pz)_3_Cu(PCN)] (compound B; HB(pz)3 stands for tris(pyrazolyl)borate, PCN stands for tris-(cyanoethyl)phosphane, gold NPs) were employed. The results indicated that the drug loading achieved 90% in case of gold NPs-A and 65% in case of gold NPs-B. For gold NPs-A conjugated systems, a release study in water solution was conducted over 4 days, which displayed a slow release of up to 10% [138].

The utilization of Au NPs capped with L-cystine methylester hydrochloride, combined with the incorporation of p53 plasmid DNA, leads to the increase of p53 expression levels, induction of apoptosis in A549 cells, and ultimately hinders their proliferation. This approach does not result in any adverse cytotoxic effects on normal lung cells [139].

Magnetic nanoparticles are frequently utilized as carriers in the development of targeted delivery systems for anticancer drugs. This preference is not only attributed to their magnetic vector potential, but also due to their ability to incorporate additional therapies and diagnostic processes. Plasmonic nanoparticles are similarly favored as carriers in targeted delivery systems for comparable reasons.

## 7. INPs in Antiviral Therapy

### 7.1. INPs Antiviral Application

Some metal based INPs are effective antiviral agents even when used in their original form. In this way, antiviral activity is possessed by NPs of silver, gold, and zinc, titanium, and iron oxides [140,141,142].

The exact mechanism of action of INPs on viruses has not yet been determined, however, it is supposed to involve several different simple mechanisms. It is proposed that, metal or metal oxide NPs interfere with the functions of viral enzymes, similar to conventional antiviral drugs. Furthermore, the mechanisms by which viral particles are degraded/inactivated by NPs may also include the breaking of disulfide bonds (which maintain the structure of viral particles) [143] or the release of metal ions. The interaction between viral envelopes and metal ions results in the formation of ROS. These ROS, which contain unpaired electrons, are known for their instability and quick reaction with biomolecules in electron exchange reactions. As a result, oxidative damage occurs, leading to changes in the structure of key biological macromolecules such as polysaccharides, proteins, lipids, and nucleic acids in viruses [144]. The prevention of viral attachment and entry into host cells can be achieved through the binding or disruption of viral surface structures such as spike glycoproteins by NPs. In therapeutic applications, the use of NPs can also assist in enhancing the host’s antiviral immune response and hinder the activity of viral enzymes [145]. Photodynamic and photocatalytic nanomaterials (like TiO_2_ NPs or coatings) can provide effective broad-spectrum and long-lasting pathogen kills (due to photocatalytic production of ROS) and offer a potential way to sterilize surfaces when exposed to light [146]. Multiple groups have showed photocatalytic virus inactivation activity of TiO_2_ coating materials. The TiO_2_ showed virucidal efficacy against human norovirus and a few norovirus surrogates (murine norovirus, bacteriophage MS2, and feline calicivirus) [147]), human influenza A (A/PR8/H1N1) [148], and herpes simplex virus 1 (HSV-1) [149].

Silver NPs are commonly regarded as an antiviral agent compared to other metals and their oxides. Research has indicated that silver NPs of sizes 2–15 nm strongly inhibited SARS-CoV-2 at concentrations between 1 and 10 ppm, with a noticeable cytotoxic effect above 20 ppm. Furthermore, the luciferase-based pseudovirus entry assay demonstrated that silver NPs significantly impeded viral entry by disrupting the virus’s integrity [150]. Porcine epidemic diarrhea virus could be suppressed by Ag nanorods coated with Au. Their mode of action in antiviral activity involves hindering the entry of the virus and reducing the mitochondrial membrane potential and caspase-3 activity [151]. The PVP-functionalized Ag NPs were also found to exhibit antiviral properties against respiratory syncytial virus as well as immunomodulatory activities [152].

The ability of silver NPs to interact with viruses can be modified by producing them in various sizes. The Ag-chitosan composites including Ag NPs with different average diameters (3.5, 6.5, and 12.9 nm) were prepared in [153]. As the amount of Ag NPs increased in the Ag-chitosan composites, the antiviral activity against H1N1 influenza A virus increased for all tested sizes of Ag NPs. Neat chitosan was found to be devoid of antiviral activity, indicating the indispensability of Ag NPs for the antiviral activity of these composites. In contrast, composites comprising smaller Ag NPs manifested stronger antiviral activity at equivalent concentrations of Ag NPs. The size of Ag NPs also plays a significant role in their effectiveness against HSV-2. In a study conducted by P. Orlowski, the interaction between tannic acid-modified Ag NPs and the glycoprotein spikes on the virion surface of the virus was investigated [154]. The microscopy analysis revealed that these spikes had a center-to-center spacing ranging from 9 to 13 nm and a height varying from 10 to 25 nm. The results of the study showed that the smaller Ag NPs (13 nm) exhibited a greater binding efficiency compared to the larger ones (33 and 46 nm).

Green synthesized by means of *Glaucium flavum* leaf extract Au NPs showed effective antiviral function against Influenza A/Puerto Rico/8/34 (H1N1) virus [141]. The 50% inhibition of viral replication was observed at Au NPs concentration of 210 μg/mL. Enhancing the concentration to 250 μg/mL led to the attainment of 60% antiviral activity [144].

The antiviral activity of bare ZnO NPs with a diameter of 20–50 nm and PEG-modified ones with a size ranging from 16–20 nm, against influenza H_1_N_1_ virus, was evaluated. The maximum biocompatible concentrations of bare ZnO NPs (75 ppm) and PEGylated ZnO NPs (200 ppm) demonstrated viral inhibition of 52.2% and 94.6%, respectively. Reduction in H_1_N_1_ occurred only when the virus was internalized in host cells [155]. The ZnO NPs with hexagonal and spherical shapes, measuring 11.50 nm, were observed to interact with host cell angiotensin-converting enzyme2 receptors, which prevented the entry of SARS-CoV-2 viruses as well as inhibiting the RNA replication and protease activity of said viruses in lung fibroblast cells [156].

The replication process of Newcastle disease virus in eggs was restrained by spherical TiO_2_ NPs with a concentration between 6.25–100 μg/mL, as determined by the hemagglutination assay. The likely mechanism for this inhibition involves the impairment of the glycoproteins’ spikes followed by the interception of viral attachment to host cells [157].

Antiviral properties have been observed for iron oxide NPs. A study conducted on silica showed that both Fe_2_O_3_ and Fe_3_O_4_ NPs can modify the conformation of HCV glycoproteins (E1 and E2) and the spike protein’s receptor binding domain (RBD) of SARS-CoV-2. Furthermore, Fe_3_O_4_ NPs can build a stable complex with SARS-CoV-2 RBD and Hepatitis C E1 and E2 [158]. It has also been reported that iron oxide nanozymes (200 nm) can neutralize influenza A viruses by creating ROS, which promote viral lipid envelope peroxidation [159].

### 7.2. INPs-Based Nanomaterials for Antiviral Drug Delivery

Gold and silver NPs have been widely employed for the purpose of the controlled release of drugs against viral infections.

Oseltamivir-modified Ag NPs with antiviral properties against influenza H_1_N_1_ influenza was obtained in [160]. In comparison to free oseltamivir and silver, the Ag NPs modified with oseltamivir exhibited a remarkable ability to hinder the H_1_N_1_ infection. Through experiments conducted on Madin–Darby canine kidney cells, it was determined that the Ag oseltamivir hybrid systems effectively prevented DNA fragmentation, chromatin condensation, and the activity of caspase-3. Furthermore, the systems prevented the accumulation of ROS by the H_1_N_1_ virus and the activation of protein kinase B and p53 phosphorylation, indicating their strong antiviral activity.

Gold NPs were considered as carriers for loading drugs (abacavir and lamivudine) for human immunodeficiency virus (HIV) treatment [161]. To obtain the prodrug candidate, the primary hydroxy groups of the drugs were functionalized with 11-mercaptoundecanoic acid. This resulted in an ester group that can be easily hydrolyzed, allowing the drug to be released from the Au NPs through enzymatic or pH-mediated hydrolysis. The drugs were released in acidic conditions and exhibited similar IC_50_ values as the free drugs (less than 10 µM) in cellular assays, indicating their ability to inhibit viral replication.

The MNPs can be used as carriers not only for antitumor, but also for antiviral therapy. Spherical γ-Fe_2_O_3_@SiO_2_-zidovudine MNPs with a core–shell structure and with an average diameter of 25 nm were obtained for magnetic guided drug targeting and biological application in [162]. The anticancer and cytotoxic properties of these MNPs surpassed those of the drug zidovudine by several orders of magnitude. Additionally, Fe_3_O_4_/NH_2_-Ag was employed as a superparamagnetic nanohybrid carrier for acyclovir, whereby the material offered controlled delivery and sustained release behavior lasting for several hours. The kinetics of drug release depended on pH [163].

By coupling a triazine-based dendrimer onto a magnetic nanomaterial, a Fe_3_O_4_@SiO_2_@TAD-G3 dendritic nanostructure was generated [164]. The resulting product was loaded with two drugs, Favipiravir and Zidovudine, and boasted a substantial drug-loading potential. Totals of 63.2% of Favipiravir and 76.5% Zidovudine were absorbed by the dendritic structure. Under pH conditions of 1.5 and 6.8, Fe_3_O_4_@SiO_2_@TAD-G3 released 90.8% and 80.2% (for Favipiravir) and 95.5% and 83.4% (for Zidovudine), respectively, over 600 min at a temperature of 37 °C.

Glycine-modified magnetic nanoparticles, ranging in size from 10 to 15 nm, showed activity against the pandemic influenza strain A/H_1_N_1_/Eastern India/66/PR8-H_1_N_1_. In experiments, 50% cell viability (TD50) was observed when administering 4.25 ± 0.2 pg of IONPs [165]. The NPs were able to inhibit the replication of the influenza H_1_N_1_ virus at the transcript level of viral RNA, with as little as 7 pg/mL of FeO NPs within a 24-h timeframe, reducing the viral genomic copies by 100-fold.

Graphene oxide and chitosan functionalized IONPs has potential to be used against coronaviruses. The observed effect is connected both with the exploitation of graphene oxide and chitosan. Analyses of the receptor-binding domain of SARS-CoV-2 and the binding of human angiotensin-converting enzyme 2 through the surrogate virus neutralization test indicated inhibition of SARS-CoV-2 virus (86%) for synthesized nanostructures [166].

Previously covered examples of antiviral drugs delivery systems are shown in Table 3.

When it comes to antiviral treatment, inorganic nanoparticles are deemed as potent agents for eradicating viruses while also serving as a useful drug carrier. Consequently, the selection of a suitable drug carrier in this scenario is based on its antiviral efficacy.

## 8. The Effect of Protein Corona on Targeted Theragnostic Nanosystems

The successful creation of functional theragnostic nanosystems is associated not only with the accumulation of agents in target tissues and the subsequent performance of their function for the prolonged release of therapeutic molecules, and heat generation or imaging. It is also necessary to reduce their accumulation outside the target and excrete them from the body. These complications are related to a phenomenon called “protein corona” (PC) formation that occurs when NPs interact with different types of plasma proteins [167,168].

Proteins, such as immunoglobulin G (IgG) and fibrinogen, give therapeutic nanosystems new physicochemical properties different from the original ones, and therefore change the therapeutic effect. This process changes their intended functionality and biological fate. In this way, the PC can influence highly specific biological complexes such as exosomes [169] and viruses [170] through unpredictable tissue distribution. The PC has a dynamic composition that shifts over time. Comprehending the formation of the biocorona is crucial in predicting the conduct of NPs in biological applications, such as nanotoxicology, and for creating nanoscale drug delivery platforms.

The creation of a PC is contingent upon the physical and chemical traits of both NPs and their biological setting [171]. As different NPs may be administered through varying routes, the composition of the PC fluctuates depending on the sort of biological fluid (e.g., blood, pulmonary, or intestinal) and protein concentration [172]. The characteristics and composition of PC are impacted by a range of physicochemical properties of NPs, including size, shape, morphology, surface chemistry, surface charge, conjugated targeting ligands, and coatings [173,174].

Two methods can be implemented to prevent undesired effects linked to PCs. First, the NP surface can be endowed with particular functionalities by incorporating diverse chemical groups that serve to conceal the NP from the detection of immune cells. Second, application of a polymer coating, such as PEG, (a.k.a. PEGylation), can hinder detection of the NP by the reticuloendothelial system [175].

The PC should not be viewed as something to avoid at all times, as it is considerably impacted by certain ailments in patients from whom the plasma is derived. As a result, the individual PC has emerged as a compelling method to screen protein biomarkers in plasma for the timely identification and management of cancer ailments. This approach could have a significant impact on the field of cancer immunotherapy, as evidenced by recent research [176]. The combination of checkpoint inhibitors with radiotherapy forms the basis of these therapies. The joint treatment triggers the production of pro-inflammatory proteins induced by radiation, leading to immune stimulation. Additionally, it elevates the exposure of cancer-specific antigens by releasing them from cancerous cells that die due to radiotherapy [177,178]. Consequently, NPs may be utilized to capture these antigens, which are linked to the tumor, and convey them to antigen-presenting cells to stimulate anti-cancer immunity [179].

It is possible that after using the PC to reach the target cells, it will not interfere with the therapeutic effect of the nanoparticles and associated therapeutic molecules. A study by Hahn et al. shows the possibility of selective separation of carboxyl-functionalized polystyrene NPs and their overlying PC (produced from the murine plasma proteins) after cellular uptake by means of endosomal sorting [180].

Further exhaustive research needs to be conducted to establish the connection between the shape, modification of nanoparticles, their pathways in the body, and the attributes of the protein crown created on them. Another crucial area of study is how the PC interacts with the immune system and the cells in targeted tissues. These outcomes will lay the foundation for the creation of advanced nanomaterials that possess personalized PCs. This new strategy will overcome the previous hindrances related to PCs and transform them into valuable assets.

Moreover, the acquired information will make a major contribution to the enhancement of the effectiveness of targeted drug delivery systems, along with magnetic agents for diagnosing and treating tumor diseases. The knowledge obtained will also serves as significant stimulus for the advancement of nano-immunotherapy.

## 9. Conclusions

The use of INPs raises great hopes in terms of the effectiveness of antiviral and anticancer treatment and cancer early diagnosis. There are numerous possibilities for their use. Drug delivery based on INPs allows a wide variety of substances (chemotherapeutic and antiviral drugs, nucleic acids, peptides etc.) to be transported and gradually released in a targeted tissue. Such hybrid drug-based nanosystems will reduce the toxicity of drugs for healthy organs; facilitate the administration of drugs by reducing the number of their uses, and to reduce the oscillations of drug molecules concentration acting on the affected organ. Combining drug delivery with HT as an adjuvant therapy can enhance the desired anticancer effects, while INPs may facilitate local HT alone specifically utilizing MNPs or plasmonic NPs. Laser-activated interaction of Au-NPs with tumor is a platform for photodynamic therapy enhancement. The treatment process can be tracked by MRI imaging with high accuracy, due to the presence of magnetic theragnostic agents directly in the targeted tissues. The USSPIONPs will possibly improve the efficiency of early cancer detection without exhibiting high toxicity as contrast agents based on complexes of Gd^3+^ ions.

**Figure 4:**
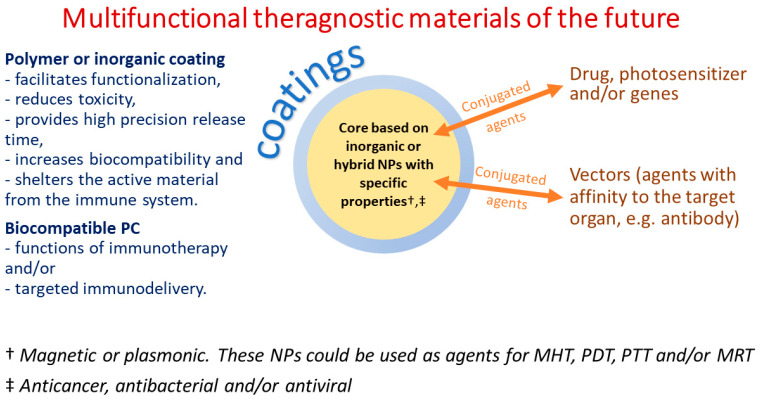
Generalized scheme of multifunctional theragnostic materials.

One characteristic of incorporating NPs and nanotechnology in medicine and pharmacology is the interdependence between the advancement of treatment and diagnosis. Instead of operating separately, these areas are highly interconnected and synchronized. Beyond merely using the resulting material across various sectors, highly intricate and intelligent drug therapy substances are emerging and becoming refined (Figure 4). Consequently, multiple forms of therapy and diagnostics can be combined, and treatment efficiency can be carefully monitored [27,28,181,182,183,184].

Such systems contain NPs with different properties (magnetic or plasmonic), which, due to their properties, can be used as agents for MRI, MHT, and PDT. Furthermore, these NPs should have a high surface area that can be modified with various coatings and agents. Hybrid inorganic NPs can serve as the core. The combination of various inorganic materials will make it possible to give magnetic systems more properties and, accordingly, more functions. Additional possibilities (targeted delivery and treatment) will be provided by conjugated agents, which may also be of more than one type. The use of various functional coatings will expand the functionality of future drug systems and increase their efficiency. The creation of such systems is a complex but interesting challenge for today’s researchers, which will allow us to create drugs of the future with increased efficiency and lower toxicity (than several different drugs with different functionality).

However, the development of complex systems and their precursors is not as fast as desired.

## Figures and Tables

**Table 1:** Potential MRI contrast agents.

MRI Agent Core Composition	NPs Size/Shape	Type of Contrast Agent	Vector (Targeting Agent)	Application Test	Reference
Fe_3_O_4_-Au	25 nm octahedral-shaped	T_2_	EPR, passive	in vitro (4T_1_ cancer cell line); in vivo (breast cancer model)	[27]
Fe_3_O_4_-Au mixed	Au 2 nm; Fe_3_O_4_ 15 nm	T_2_	EPR, passive	in vivo (glioblastoma)	[28]
MIONPs	20 nm	T_2_	HER2 single-chain antibody	in vitro (NCI-N87 human gastric cancer cells and human pancreatic cancer cells SUIT2); in vivo (pancreatic cancer)	[29]
MIONPs		T_2_	BCZM	in vivo (breast cancer cells transfected with the VEGF-165 isoform)	[30]
Au coated Fe_3_O_4_	46 nm	T_2_	C225	in vitro and in vivo (human glioma)	[31]
Au coated Fe_3_O_4_	50 nm	T_2_	Prostate stem cell antigen antibody	in vivo (prostate tumors)	[32]
MIONPs		T_2_	anti-epidermal growth factor receptor antibody	in vivo (lungs tumor)	[33]
Fe_3_O_4_	22 nm	T_2_	arginine-glycine-asparticacid-tumornecrosis factor-related apoptosis-inducing ligand	in vivo (colorectal cancer)	[34]
Fe_3_O_4_/mesoporous silica-Au	16 nm	T_1_ and T_2_	peptide cyclo[Arg-Gly-Asp-D-Phe-Lys]	in vivo (fibro-sarcoma)	[35]
MIONPs	10 nm	T_2_	glypican-3 (GPC3)-specific aptamer (AP613-1)	in vitro and in vivo (liver cancer)	[37]
Fe_3_O_4_-Au	Fe_3_O_4_ 2 nm Fe_3_O_4_-Au 7 nm	T_1_ and T_2_	tumor homing peptide (LyP-1)	in vitro and in vivo (hepatocellular carcinoma)	[38]
MIONPS		T_2_	folic acid	in vivo (lungs cancer)	[39]
MIONPS	1–3 nm	T_1_	-	in vivo (mice)	[42]
Co_1−x_Mn_x_Fe_2_O_4_	10–50 nm	T_2_	-	in vivo (mice)	[45]

**Table 2:** Anticancer targeted drug delivery systems.

**INPs Core**	**NPs Size/Shape**	**Covering**	**Drug Substance**	**Application Tests**	**Reference**
Fe_3_O_4_	9 nm	PECA	Cisplatin and Gemcitabine	In vitro experiments of drug release and magnetic mobility	[115]
Fe_3_O_4_	6 nm	chitosan-polyacrylic acid	5-FLU	In vitro drug loading and release studies	[116]
Flower-shaped magnetite	16 nm	poly (N-vinylcaprolactam-co-acrylic acid)	Doxorubicin4	In vitro drug release (pH and temperature dependent). Cytotoxicity assay and cellular uptake study: MCF-7 (breast cancer cell line) and A375 (melanoma cell line)	[117]
MIONPs	10–20 nm	Pluronic F127 and branched polyethylenimine	Doxorubicin	In vitro cellular uptake studies (HepG2)	[118]
Fe_3_O_4_	13 nm	silica and covalently modified with [(3-triethoxysilyl)-propyl]-succinic acid–polyethylene glycol	Doxorubicin	Cytotoxicity Assay (epithelial, human breast cancer cell—MDA-MB231, HepG2, animal model for stage IV human breast cancer-4T1, colon carcinoma CT26, and melanoma—B16)	[119]
Fe_3_O_4_	25–40 nm	chitosan	Oxaliplatin, Irinotecan	-	[120]
MIONPs		PLGA	5-FLU	pH-dependent release of 5-FLU	[121]
Fe_3_O_4_	1–10 nm	polyarginine hydrochloride, DEX	Doxorubicin	In vivo and ex vivo biodistribution studies; Flow cytometry (MCF-7)	[122]

**Table 3:** Antiviral targeted drug delivery systems.

INPs Core	NPs Size/Shape	Covering	Drug Substance	Virus	Reference
Ag	2–3 nm		Oseltamivir	influenza H_1_N_1_	[160]
Au	3 nm	Glucose	Abacavir and Lamivudine	HIV	[161]
γ-Fe_2_O_3_	25 nm	SiO_2_	Zidovudine	-	[162]
Fe_3_O_4_@SiO_2_/NH_2_-Ag	150–400 nm	-	Acyclovir	-	[163]
Fe_3_O_4_@SiO_2_	15–35 nm	Dendrimer	Favipiravir and Zidovudine	-	[164]
Fe_3_O_4_	10–15	Glycine	Glycine	influenza H_1_N_1_	[165]
Fe_3_O_4_	25 nm	Graphene oxide and chitosan		SARS-CoV-2	[166]

## Data Availability

No new data were created or analyzed in this study.
